# Are behavioral interventions effective in increasing physical activity at 12 to 36 months in adults aged 55 to 70 years? a systematic review and meta-analysis

**DOI:** 10.1186/1741-7015-11-75

**Published:** 2013-03-19

**Authors:** Nicola Hobbs, Alan Godfrey, Jose Lara, Linda Errington, Thomas D Meyer, Lynn Rochester, Martin White, John C Mathers, Falko F Sniehotta

**Affiliations:** 1 Institute of Health & Society, Newcastle University, Baddiley-Clark Building, Richardson Road, Newcastle upon Tyne, NE2 4AX, UK; 2 Clinical Ageing Research Unit, Newcastle University, Campus for Ageing and Vitality, Newcastle upon Tyne, NE4 5PL, UK; 3 Human Nutrition Research Centre, Institute for Ageing and Health, Newcastle University, Biomedical Research Building, Campus for Ageing and Vitality, Newcastle upon Tyne, UK; 4 Walton Library, Newcastle University, Newcastle upon Tyne, NE4 5PL, UK; 5 Institute of Neuroscience, Newcastle University, Ridley Building, Newcastle upon Tyne, NE1 7RU, UK; 6 Fuse, UKCRC Centre for Translational Research in Public Health, Institute of Health & Society, Newcastle University, Baddiley-Clark Building, Richardson Road, Newcastle upon Tyne, NE2 4AX, UK

**Keywords:** Physical activity, intervention, aging, systematic review, meta-analysis

## Abstract

**Background:**

Retirement represents a major transitional life stage in middle to older age. Changes in physical activity typically accompany this transition, which has significant consequences for health and well-being. The aim of this systematic review was to evaluate the evidence for the effect of interventions to promote physical activity in adults aged 55 to 70 years, focusing on studies that reported long-term effectiveness. This systematic review adheres to a registered protocol (PROSPERO CRD42011001459).

**Methods:**

Randomized controlled trials of interventions to promote physical activity behavior with a mean/median sample age of 55 to 70 years, published between 2000 and 2010, were identified. Only trials reporting the long-term effect (≥ 12 months) on objective or self-reported physical activity behavior were included. Trials reporting physiological proxy measures of physical activity were excluded. Meta-analyses were conducted when trials provided sufficient data and sensitivity analyses were conducted to identify potential confounding effects of trials of poor methodological quality or with attrition rates ≥ 30%.

**Results:**

Of 17,859 publications identified, 32 were included which reported on 21 individual trials. The majority of interventions were multimodal and provided physical activity and lifestyle counselling. Interventions to promote physical activity were effective at 12 months (standardized mean difference (SMD) = 1.08, 95% confidence interval (CI) = 0.16 to 1.99, pedometer step-count, approximating to an increase of 2,197 steps per day; SMD = 0.19, 95% CI = 0.10 to 0.28, self-reported physical activity duration outcome), but not at 24 months based on a small subset of trials. There was no evidence for a relationship between intervention effectiveness and mode of delivery or number of intervention contacts; however, interventions which involved individually tailoring with personalized activity goals or provision of information about local opportunities in the environment may be more effective.

**Conclusions:**

Interventions in adults aged 55 to 70 years led to long term improvements in physical activity at 12 months; however, maintenance beyond this is unclear. Identified physical activity improvements are likely to have substantial health benefits in reducing the risk of age-related illnesses. These findings have important implications for community-based public health interventions in and around the retirement transition.

## Background

Physical inactivity is a leading cause of death and disease. Epidemiological evidence shows a relationship between physical activity (PA) and reduced risks of coronary heart disease, Type II diabetes and some cancers, as well as increasing life expectancy [1,2]. The prevalence of disease and disability increases with age, making PA promotion an important public health objective to mitigate the burden of age-related illness [2,3]. However, over 50% of adults in Europe and the USA do not achieve public health recommendations for levels of PA [4-6].

PA is a modifiable behavior that varies in relation to major life events and transitions [7,8]. Retirement represents a key transition which impacts on physical and social activities [9]. Cross-sectional and longitudinal cohort studies show that PA levels change during retirement; however, the direction and magnitude of changes are inconsistent [10-13]. Previous occupation, socioeconomic and social factors may be important determinants of PA levels during retirement [14-18]. As PA levels are susceptible to change in retirement, then the retirement transition represents an ideal opportunity to intervene to increase and sustain PA behavior and, in turn, encourage healthy aging.

We are interested in promoting PA behaviors of people in and around the retirement transition. The average age of retirement varies between nations and from one year to the next. Since the early 2000s, retirement age is increasing in industrialized nations [19]. In the UK between 2004 and 2010, the average age of retirement rose from 64 to 65 and 61 to 62 for men and women, respectively [20]. Thus, in order to investigate the effect of interventions to increase PA in adults of a likely retirement age, we are focusing on adults between the ages of 55 and 70 years. Evidence from systematic reviews of PA interventions in middle-aged to older adults show moderate effects of interventions on PA behavior in the short- to mid-term (mean effect size of 0.28 [21] and 0.19 [22]). More effective interventions identified by these reviews were interventions which provided professional guidance and on-going support [21] and behavioral rather than cognitive interventions [22]. However, the evidence of PA interventions in the long-term effect is limited with only a few studies with follow-up assessments beyond 12 months [22]. From a public health perspective, it is critical to know whether PA behavior change can be sustained in the long-term. This systematic review and meta-analysis aims to synthesize the evidence from randomized controlled trials (RCT) on the effectiveness of interventions to promote long-term PA change (≥ 12 months) in adults aged 55 to 70 years.

## Methods

### Study selection criteria and search strategy

This systematic review adheres to a registered protocol [23] (see Additional file 1). Only RCTs of interventions assessing and reporting PA behavior ≥ 12 months after randomization were included. Included trials assessed PA behavior using objective or self-report measures. Interventions were compared to a no-intervention, minimal or usual care intervention; or a different type of intervention. Included trials studied healthy participants or those 'at risk' of chronic disease with a mean or median age of 55 to 70 years. 'At risk' participants were reported as having at least one of the following disease risk factors: hypertension, impaired glucose tolerance, overweight/obese, hyperlipidaemia, dyslipidaemia, family history, metabolic syndrome or osteopenia. Publications of any language with an English language abstract and with a country of origin of one of the 'most developed countries' within the United Nations index [24] were considered for inclusion.

Trials with inadequate randomization were excluded as were trials involving participants who were institutionalized or recruited on the basis of taking a particular medication or having a pre-existing chronic or acute medical condition. Trials that only reported PA behavior earlier than 12 months after randomization, that only reported physiological proxy measures of PA as distinct from PA behavior, were laboratory-based exercise studies, or promoted high or elite performance training were also excluded.

Twelve electronic databases were searched for articles published between January 2000 and November 2010 (Medline; Embase; PsycInfo; Scopus; Web of Science; CINAHL; ASSIA; Cochrane Database of Systematic Reviews, CAB Abstracts, Conference Papers Index, WorldCat Dissertations database and Index to Theses). Search terms relating to PA, middle- to older-aged people and RCT were translated into a Medline search strategy (see Additional file 2), which was adapted for other databases (available on request). Reference lists of reviews of PA interventions were hand-searched. After removing duplicate publications, the title and abstract of each publication was screened independently by two reviewers. When eligibility could not be ascertained or when reviewers disagreed, the full text was screened. Full text publications were also screened by two reviewers (Kappa = 0.91) and a third reviewer was consulted to resolve discrepancies. Reference lists of included publications were searched for additional publications.

### Data extraction

Data from each included publication were extracted by one reviewer and independently checked by another (Kappa = 0.86); a third reviewer was consulted to resolve discrepancies. Authors were contacted for missing data and to provide additional intervention material. Intervention content was coded in line with intervention reporting guidance [25] to identify the modes of delivery and intervention intensity and trial quality was assessed using the Cochrane risk of bias tool [26].

### Data analysis

The primary outcome was PA behaviors. Intervention effects were assessed by grouping trials for meta-analysis by the method of assessing PA and according to the duration of follow-up measurement after randomization. Random effects models (Review Manager (RevMan), version 5.1, Copenhagen, Denmark) were used and standardized mean differences (SMD) or odds ratios (OR) were calculated depending on whether the measurement scale was continuous or dichotomous. When trials included multiple intervention arms, which compared a PA intervention arm with a dietary intervention arm, the PA intervention arm was compared to the 'no intervention, control' arm. When trials included multiple intervention arms, which compared different types of PA intervention, the arm with the most intensive intervention content was compared with the 'no intervention, control' arm. The most intensive intervention arm was defined as the arm with the greater number of 'active' intervention contacts, that is., contacts when the intervention was delivered rather than contacts when only measurements were taken. When the number of contacts was equal in the intervention arms, the most intensive arm was defined as the arm delivered by a human rather than an automated system; the arm providing the most different types of information, using the most different modes of delivery or intervention aids; or the arm with the most intense exercise prescription. The content of all intervention arms reported in a trial is described in Additional file 3.

Data from intention-to-treat analysis were used when reported. If trials assessed PA using multiple methods, data were included in each corresponding meta-analysis. Estimations for total PA were selected for analysis over specific PA domains (for example, total PA rather than leisure time PA). Authors were contacted to provide mean and standard deviation when only the median and range were reported. When trials reported change scores from baseline, final values were computed where possible or requested from authors. For each main-effect analysis, sensitivity analyses were conducted by excluding trials of poor methodological quality or with attrition rates ≥ 30%. Possible publication bias was assessed using funnel plots and Egger's tests [27]. Narrative analyses were applied when trials did not report sufficient data for meta-analyses or when the method of PA assessment was not equivalent to that used in any other trial.

## Results

**Figure 1:**
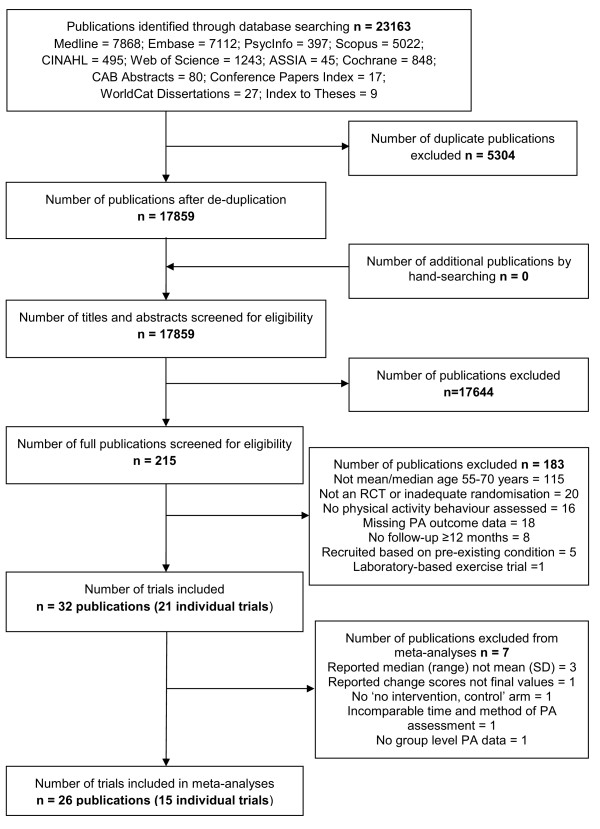
**Trial selection flow diagram adapted from PRISMA **[80].

Thirty-two publications were included which reported on 21 distinct trials. Of these 21 trials, 15 were included in the meta-analyses [28-42] and six trials were analyzed narratively [43-48]. Nine trials were reported in multiple publications. The Green Prescription trial [29] was reported in two other publications [49,50]; the Community Health Advice by Telephone trial [30] in one other publication [51]; the Woman on the Move through Activity and Nutrition trial [31] in one other publication [52]; the Women's Lifestyle Study [32] in one other publication [53]; the Finnish Diabetes Prevention Study [33] in three other publications [54-56]; the Keep Active Minnesota trial [34] in one other publication [57]; the trial by Opdenacker and colleagues [36] in one other publication [58]; the Vitalum trial [39] in one other publication [59]; and the Pre-diabetes Risk Education and Physical Activity Recommendations and Encouragement trial [42] in one other publication [60]. Figure 1 displays the number of publications included and excluded at each stage, and reasons for exclusion (see Additional file 4 for PRISMA checklist). The characteristics of included trials including a description of the population, intervention and outcomes are tabulated (see Additional file 3).

Five authors provided additional unpublished data needed for meta-analyses, including mean and standard deviation when only the median and range were published and final values when only change scores from baseline were published [29,31,32,41,42]. Twelve authors responded to the request for additional intervention material [28,29,32,34,37-41,43-45], seven of whom provided additional material [28,29,34,39,40,44,45]. Unpublished material provided in languages other than English was translated into English using online services and/or native speakers [28,39,45]. Additional English language publications describing intervention content were obtained for six trials [61-66].

Sixteen interventions were delivered by health professionals [28-38,42,44-47], one intervention by the researcher [39], one by the participant under instruction (that is, self-help) [40], and the intervention provider was unclear in three trials [41,43,48]. The delivery format was multimodal for 14 trials (that is, face-to-face individual basis and via the telephone and/or printed material [29,30,32,43,44,47]; face-to-face group basis and via the telephone and/or printed material [34,45,46]; face-to-face individual and group basis plus via the telephone or printed material [33,36,38]; via the internet and printed materials [41]; or via the telephone and printed material [39]); unimodal for four trials (that is, face-to-face individual only [28]; face-to-face group only [31,42]; or printed material only [40]); and it was unclear whether the format was face-to-face individual or group for three trials [35,37,48].

The intervention settings included healthcare premises [29,32,33,38,42,43,45,47], the participant's home [30,34,36,39-41], healthcare premises and at home [28,35], in a university facility [48], in a community setting [46] or this information was unspecified [31,37,44]. On average, trial length was 17 months from randomization (SD = 6.6), the 'active' intervention period was 8 months (SD = 4.6; range 1 to 11) with 37 contacts (SD = 60; range 1 to 228). The intervention period and the number of contacts were not specified in one trial [38]. Trials were conducted in the USA [30,31,34,35,37,38,46,48], Belgium [36], The Netherlands [39-41,44,45], UK [42], Finland [33], New Zealand [29,32], Japan [28], Australia [43] and Canada [47]. Six trials used a clustered RCT design and accounted for clustering in analyses [29,38,40,41,44,47].

All of the interventions, except one which involved group education and prescribed a standard PA goal of 150 minutes per week of moderately intense PA [31], were individually tailored to some degree. Five interventions provided participants with individually tailored exercise prescriptions, tailored on the basis of target heart rate [35,45,48], submaximal VO_2max _step test results [47] and baseline total energy expenditure [37]. The remaining interventions provided information specific to the individual to match their potential or actual health risk, their environment and local opportunities, and/or their individual PA goals. Thirteen of 21 interventions employed core self-regulation principles, such as goal setting, planning, self-monitoring and providing feedback [28-30,32-34,36,39-44].

In total, trials reported on 10,519 participants, 61% of whom were female. The mean age of participants was 60.7 years (SD = 4.4; range 55 to 67.6). Two trials provided the intervention cost per participant [28,29] but none reported a cost-effectiveness evaluation. Two trials specifically targeted participants in the retirement transition, namely retired employees [36] and recent retirees from a retirement workshop [41].

For many trials, there was insufficient information to permit conclusive judgements about methodological quality; the risk of bias was unclear in approximately 50% of possible judgements. Where judgements could be made, approximately 25% of trials were rated as poor quality attributable to a lack of blinding of participants or intervention personnel, missing outcome data or selective outcome reporting (see Additional file 5). Mild asymmetry was evident in the funnel plots; however, the results of Egger's test were not statistically significant (see Additional file 6).

### PA outcomes

Six trials assessed PA objectively: five trials used pedometers deriving step-count [28,31,35,36,42] and one trial used an accelerometer deriving vector magnitude [36]. Twenty trials estimated PA duration by self-report questionnaires reported as minutes of PA or energy expenditure (that is, kcal, kJ or metabolic equivalent (MET)) [29-48]. Four trials assessed PA using both objective and self-report methods [31,35,36,42]. Of the 15 trials included in the meta-analyses, four trials reported pedometer step-count at 12 months [28,35,36,42]; 11 trials reported PA duration at 12 months using a continuous measurement scale [29,30,32,34-37,39-42]; three trials reported PA duration at 12 months using a dichotomous measurement scale of the percentage of participants meeting a target PA duration [32,33,38]; two trials reported PA duration at 18 months using a continuous measurement scale [31,39]; and four trials reported PA duration at 24 months using a continuous measurement scale [32,34,36,41]. Seven trials were included in more than one meta-analysis [32,34-36,39,41,42].

#### Pedometer step-count

**Figure 2:**
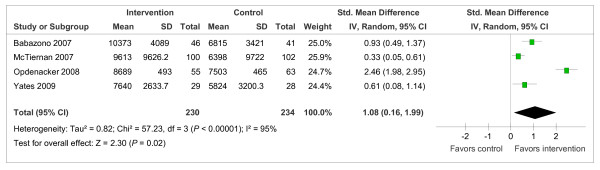
**Trials reporting pedometer step-counts (steps/day) at 12 months**.

When compared with controls at 12 months, interventions had a significant positive effect on step-count (Figure 2: SMD = 1.08, 95% CI = 0.16 to 1.99; *I*^2 ^= 95%, 95% CI = 90 to 97). One additional trial [31] assessed step-count 18 months after randomization and similarly identified a significant intervention effect (SMD = 0.38, 95% CI = 0.16 to 0.60); however, this trial suffered from high attrition bias and did not report conducting intention-to-treat analysis. A single trial [36] with 24-month follow-up assessed step-count and found that the previously identified beneficial intervention effect on step-count was not sustained (SMD = -0.01, 95% CI = -0.41 to 0.40).

#### Accelerometer

The single trial which reported PA using an accelerometer [36] did not identify a detectable effect of a lifestyle intervention on vector magnitude/week at 12 months (SMD = 0.18, 95% CI = -0.18 to 0.55) or at 24 months (SMD = -0.01, 95% CI = -0.42 to 0.40).

#### Self-reported PA duration

**Figure 3:**
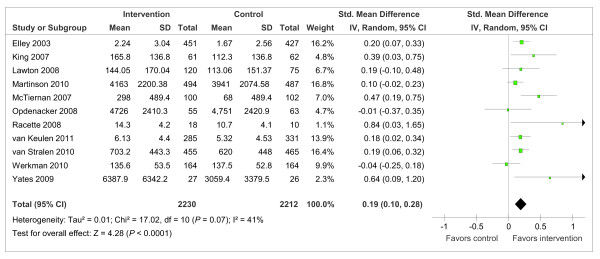
**Trials reporting duration of PA at 12 months - continuous outcome measures**.

**Figure 4:**
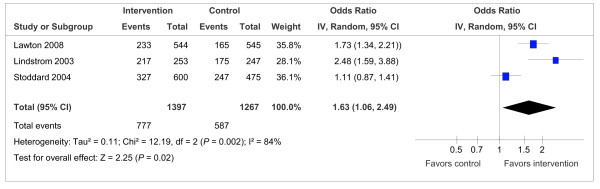
**Trials reporting duration of PA at 18 months - continuous outcome measures**.

Interventions had a significant small positive effect on PA duration at 12 months when measured on a continuous scale (Figure 3: SMD = 0.19, 95% CI = 0.10 to 0.28; *I*^2 ^= 41%, 95% CI = 0 to 74). Sensitivity analysis, removing trials with high attrition rates or low quality ratings [37,40], did not alter the effect (SMD = 0.18, 95% CI = 0.07 to 0.29, *I*^2 ^= 52%). Interventions also had a positive effect on PA duration at 12 months when measured on a dichotomous scale (Figure 4: OR = 1.63 95% CI = 1.06 to 2.49, *I*^2 ^= 84%). The three studies meta-analyzed here used scales which quantified the percentage of participants meeting a target PA duration equating to meeting current PA recommendations.

Four additional trials that could not be meta-analyzed due to insufficient data, reported inconsistent intervention effects at 12 months. One trial [43] found that the intervention group reported more vigorous activity (minutes/session) than the control group (intervention: median = 20, 25^th ^to 75^th ^percentile = 0 to 35; control: median = 0, 25^th ^to 75^th ^percentile = 0 to 15, *P *< 0.05), but not more walking (intervention: median = 30, 25^th ^to 75^th ^percentile = 10 to 60; control: median = 30, 25^th ^to 75^th ^percentile = 10 to 60). Another trial [48] reported no change in self-reported PA from baseline to 12 months using the Physical Activity Scale for Elderly in response to either a diet and high exercise intervention, diet and low exercise intervention, or a diet-only intervention. The remaining two trials reported increases in PA duration reported as MET hours/week (median difference = 13.9, 95% CI = 10.6 to 18.3) [45] and change in kcal/kg/day (mean difference = 0.42, 95% CI = 0.12 to 0.72) [47].

Beyond 12 months, there was little evidence for significant intervention effects. No intervention effect was identified on PA duration at 18 months when measured on a continuous scale (SMD = 0.10, 95% CI = -0.08 to 0.29, *I*^2 ^= 55%) and similarly, the single trial which used a dichotomous scale [44] reported no effect of an individualized stage-matched intervention on PA duration (OR = 1.21, 95% CI = 0.95 to 1.54); however, this trial was rated as poor quality. In addition, no positive intervention effect was identified by four trials assessing PA duration at 24 months using a continuous scale (SMD = 0.07, 95% CI = -0.06 to 0.20, *I*^2 ^= 27%) or by the single trial using a dichotomous scale (OR = 1.33, 95% CI = 1.03 to 1.70) [32]. In contrast to these null findings, the increase in MET hours/week identified at 12 months in response to a supervised group exercise intervention [45] was retained at 24 months in one trial (median difference = 4.1, 95% CI = 0.3 to 8.3); however, this trial could not be meta-analyzed due to missing data. One trial [33] assessed PA at 36 months and reported no change in PA duration in the intervention group but detected an increase in moderate-to-vigorous PA duration (minutes/week) (intervention: median change = 61, IQR = 33 to 168; control: median change = 6, IQR = 91 to 104).

#### Trials comparing multiple interventions

Seven trials compared multiple interventions at 12 months after randomization. The results of two high-quality trials suggest that mode of delivery is not necessarily important for intervention effectiveness. King and colleagues [30] compared telephone-assisted PA counseling by a trained health educator with telephone-assisted PA counseling by an automated telephone-linked computer system and concluded that both interventions were effective in increasing weekly PA when compared to a no intervention control (SMD = -0.07, 95% CI = -0.42 to 0.28). The large Vitalum trial of 1,600 participants [39] used a full factorial design comparing three different interventions and found that at 12 months, when compared to a no intervention control group, a tailored print intervention and a combined tailored print and motivational interviewing intervention produced improvements in self-reported PA (tailored print vs. control: SMD = 0.32, 95% CI = 0.15 to 0.48; combined tailored print and motivational interviewing vs. control: SMD = 0.18, 95% CI = 0.02 to 0.34), while a motivational interviewing-only intervention did not increase PA (SMD = 0.08, 95% CI = -0.08 to 0.23). In addition, at 18 months, PA improvements were equally produced in response to all three interventions. These trials provide evidence to support the development of less resource intensive interventions that have greater potential to be cost-effective.

The type of PA that the intervention promotes may, however, be important. A trial of retired university employees [36] found that a home-based lifestyle intervention using a pedometer produced larger increases in active transport and daily steps at 12 months than a structured intervention involving supervised exercise sessions or a no intervention control (active transport, lifestyle vs. structured: SMD = 1.19, 95% CI = 0.79 to 1.59; lifestyle vs. control: SMD = 0.60, 95% CI = 0.23 to 0.97; daily steps, lifestyle vs. structured: SMD = 2.49, 95% CI = 2.00 to 2.99; lifestyle vs. control: SMD = 2.46, 95% CI = 1.98 to 2.95). King and colleagues [46] found that an aerobic and strength training intervention resulted in greater self-reported daily energy expenditure (cal/kg/day) than a stretching and relaxation intervention; this trial only provided baseline-adjusted mean values, thus SMD cannot be calculated. A small trial of 26 participants [48] suggests that the intensity of the PA promoted is not an important factor for intervention effect; no difference in changes in PA was identified in response to a high intensity exercise prescription, a low intensity exercise prescription or a no intervention control.

Tailoring the intervention for the participant may be an important factor for producing positive intervention effects. A trial of almost 2,000 participants [40] found that when compared to a no intervention control group, an environmentally tailored intervention which provided personalized PA advice and tailored information about opportunities in the environment resulted in an increase in total PA (SMD = 0.19, 95% CI = 0.06 to 0.32), while the 'basic' intervention which only provided personalized PA advice did not produce PA improvements (SMD = 0.11, 95% CI = -0.02 to 0.25). Similarly, in a trial of participants with impaired glucose tolerance, a tailored educational intervention using personalized step goals and a pedometer was more effective than an intervention using generic time-based goals and no pedometer, when compared to a no intervention control; the pedometer group produced larger increases in step-count (tailored pedometer: SMD = 0.61, 95% CI = 0.08 to 1.14; generic no pedometer: SMD = 0.36, 95% CI = -0.18 to 0.90) and self-reported walking (tailored pedometer: SMD = 0.64, 95% CI = 0.09 to 1.20; generic no pedometer: SMD = 0.29, 95% CI = -0.28 to 0.85) [42].

#### Exploratory sub-group analysis

**Figure 5:**
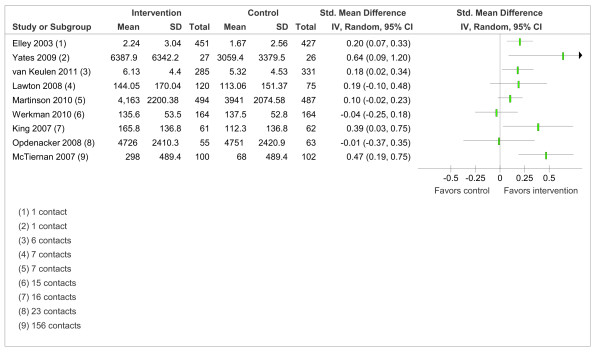
**Trials reporting duration of PA at 12 months ordered by the number of intervention contacts**.

The meta-analyses revealed heterogeneity [27]; therefore, potential reasons for this heterogeneity were probed based on intervention intensity. This secondary analysis only included trials of higher quality and was restricted to interventions on self-reported PA duration at 12 months measured on a continuous scale. Based on a median split, intervention effect was negatively associated with intervention intensity such that interventions that had more intervention contacts (≥ 11 contacts) did not have a detectable intervention effect on PA duration (SMD = 0.20, 95% CI = -0.08 to 0.47, *I*^2 ^= 71%) while interventions that had less intervention contacts (Ë‚11 contacts) had a positive intervention effect (SMD = 0.16, 95% CI = 0.06 to 0.27, *I*^2 ^= 38%). However, there was no statistically significant difference between subgroups (Chi^2 ^= 0.05, *P *= 0.82). The absence of a dose-response effect of interventions on PA duration at 12 months is displayed in a forest plot where trials are ordered by intervention intensity (Figure 5).

## Discussion

This is the first systematic review to our knowledge to synthesize evidence from RCTs focusing on the long-term effectiveness of interventions to promote PA in adults aged 55 to 70 years. We have provided evidence to show that PA interventions are effective at 12 months but this effect is not evident at 24 months. Based on a median split of the number of intervention contacts in high quality trials reporting continuous outcomes at 12 months, there is no evidence that more contacts lead to more favorable intervention effects. Evidence from trials comparing multiple interventions suggests that mode of delivery is not necessarily important for effectiveness but that tailoring the intervention to participants with personalized step-count goals or information about local opportunities in the environment may be important.

### Findings in context and interpretation

In a meta-analysis of four trials, we identified a large positive effect of 1.08 on step-count 12 months after randomization, approximating to an increase of 2,197 steps/day. The magnitude of this increase in step-count is similar to the 2,000 to 2,500 steps/day reported in previous meta-analyses of PA interventions lasting an average of 16 [67] and 18 [68] weeks. Our meta-analysis investigated the effect on step-count 12 months after randomization; therefore, this suggests that the improvements in step-count acquired in the short to medium term can be sustained into the long-term.

In a meta-analysis of 11 trials, we identified an effect size of 0.19 on self-reported PA duration 12 months after randomization. This effect is smaller than previously reported in a meta-analysis of RCT with follow-up assessments at six months (0.28) [21]. It is possible that intervention effects dissipate with time and perhaps the effects identified from trials with short or medium term follow-up assessments are not sustained in the longer-term. This hypothesis is supported by our finding that interventions were effective at 12 months but the benefits were not apparent at 24 months. Ten out of 11 trials assessing intervention effects on PA beyond 12 months consistently reported significant positive intervention effects. However, pooling trials for analysis by time and method of PA assessment did not identify positive intervention effects at 18 or 24 months. It is possible that the statistical power for each of these analyses was insufficient to detect an effect. Similarly, evidence from trials that could not be meta-analyzed did not provide clear support for positive intervention effects at 18, 24 or 36 months with most trials reporting that intervention effects has dissipated beyond 12 months while others identified positive effects in some but not all methods of PA assessment.

Another recent meta-analysis [22] that aggregated interventions with objective and self-reported PA outcomes reported an effect size of 0.19, which is the same size effect as we identified for self-reported outcomes. Our review adds to Conn and colleagues' review [22] by focusing on evidence of long-term effectiveness from RCT in adults aged 55 to 70 years. The average effect of PA interventions was higher on step-count than on self-reported PA duration. The accuracy of self-reported assessments of PA compared with objective assessments is uncertain; self-reported PA has been shown to overestimate and underestimate actual PA level [69]. Targeting specific activities, such as walking, may be more effective than targeting generic PA in people of retirement age. For example, one trial assessed PA using multiple methods and reported an increase in walking measured by a pedometer but no effect on PA measured by a self-report questionnaire or accelerometer [36].

We found a lack of evidence for a relationship between intervention effectiveness and mode of delivery or intervention intensity in terms of the number of intervention contacts, which concurs with evidence from other reviews of physical activity interventions [68,70]. However, in line with previous literature [68,71,72], there was evidence that using personalized step-count goals was better than using time-based goals, and that addressing physical environmental determinants of PA may be beneficial for PA promotion.

### Implications

The substantial effect we identified on walking could produce important health benefits for older adults, such as improving weight-related outcomes, cardiorespiratory fitness, and cognitive and psychological well-being [73-77]. Furthermore, 100 steps per minute has been proposed to represent the floor value of moderate intensity walking [78]. The intensity of walking performed in the included trials is unclear; however, if the additional 2,197 steps per day were of moderate intensity, then this equates to approximately 22 minutes of moderate PA per day for an individual, which would contribute substantially towards meeting national PA recommendations of 150 minutes of moderate activity a week [6]. Moreover, a large recent prospective study of the health benefits of PA has shown that an additional 15 minutes of moderate PA per day may be sufficient to produce health benefits [79].

### Strengths and limitations

The strengths of this review include that the search strategy was pre-specified and there was no statistical evidence that meta-analysis was affected by publication bias. Additional intervention material and data were requested from authors to improve the accuracy of the identification of intervention content and intervention intensity, and to limit missing data. We tested the sensitivity of our meta-analyses and found that the results were not influenced by trials with a high risk of bias. However, it is noted that the information provided in publications did not always allow conclusive judgements of methodological quality to be made, which resulted in many uncertain judgements. Thus, this observation limits the conclusiveness of these sensitivity analyses while highlighting the need for better reporting of trials.

This review only included trials which had been conducted with participants in countries categorized as being one of the 'most developed countries' within the United Nations index [24]. This inclusion criterion was chosen to ensure that the review focused on interventions that were applicable to populations experiencing broadly similar infrastructure, culture and standards of living. However, it is noted that, consequently, the findings of this review may not be generalizable to lower income countries. The included trials in this review had a sample mean or median age of 55 to 70 years, thus it is possible that the age of some individual participants included in this review may have been outside this bracket. However, 5 of 21 trials reported only recruiting participants within this age bracket and the average SD of the sample mean age in the remaining trials was seven years. Therefore, the relatively small variance in sample age suggests that in fact very few participants would not have been between 55 and 70 years old. In addition, the average age at retirement varies between individuals and nations, and varies over time [19]; therefore, adults aged 55 to 70 years are likely to reflect people in and around the retirement transition. Some people may have already retired, other people may be retiring soon, while other people may not have yet considered retiring.

## Conclusions

The current evidence base is limited beyond 12 months and, therefore, RCT with longer follow-up are needed. Better reporting of trials is essential for complex interventions such as those included in this review. More factorial trials are needed to identify and isolate individual intervention components, such as tailoring and environmental factors, which may be associated with effectiveness.

## Abbreviations

CI: confidence interval; PA: physical activity; RCT: randomized controlled trial; MET: metabolic equivalent; OR: odds ratio; SMD: standardized mean difference

## Competing interests

The authors declare that they have no competing interests.

## Authors' contributions

NH, AG, JL, TDM, LR, MW, JCM and FFS conceived and designed the study. LE performed the database searches. NH, AG, LR and FFS extracted the data. NH conducted data analysis and interpretation, and drafted the manuscript. AG, JL, LR, TDM, MW, JCM and FFS contributed to data interpretation. AG, JL, TDM, LE, LR, MW, JCM and FFS contributed to revising the manuscript. All authors approved the final manuscript. The corresponding author (NH) had full access to all the data in the study and had final responsibility for the decision to submit the manuscript for publication.

## Pre-publication history

The pre-publication history for this paper can be accessed here:

http://www.biomedcentral.com/1741-7015/11/75/prepub

## Supplementary Material

Additional file 1 **Registered systematic review protocol**. On inception of this systematic review, the protocol was registered with the National Institute of Health Research International Prospective Register of Systematic Reviews, PROSPERO: CRD42011001459. Click here for file

Additional file 2 **OVID Medline search strategy**. Search terms and search strategy used to search for records in the OVID Medline electronic database. Click here for file

Additional file 3 **Table of characteristics of included trials in alphabetical order by author**. Characteristics of the trials included in this review, including information on the study population, setting, physical activity outcome measures, assessment times, sample size in each intervention arm, content and delivery of intervention, and attrition rates. Click here for file

Additional file 4 **Preferred Reporting Items for Systematic Reviews and Meta-Analyses (PRISMA) 2009 checklist**. Completed PRISMA 2009 checklist detailing the page of the manuscript on which each checklist item is reported. Click here for file

Additional file 5 **Cochrane risk of bias figure**. Risk of bias present in the trials included in this review based on the Cochrane risk of bias tool. Judgements about each risk of bias item are presented as percentages across the 21 included trials. The green bars represent the percentage of trials rated as high quality (low risk of bias) on each item, the yellow bars represent the percentage of trials where judgements could not be made (unclear risk of bias) on each item, and the red bars represent the percentage of trials rated as poor quality (high risk of bias) on each item. Click here for file

Additional file 6 **Funnel plots and Egger's test results assessing publication bias in the meta-analyses**. Funnel plots and Egger's tests were used to identify the presence of publication bias in the meta-analyses. Publication bias could be tested for in the meta-analysis of trials reporting pedometer step-counts (steps/day) at 12 months; duration of physical activity at 12 months - continuous outcome measures; and duration of physical activity at 24 months - continuous outcome measures. Click here for file
