# Supplementary material for: Are behavioral interventions effective in increasing physical activity at 12 to 36 months in adults aged 55 to 70 years? a systematic review and meta-analysis
Source: BMC Med. 2013 Mar 19;11:75. doi: 10.1186/1741-7015-11-75 (PMC3681560; doi:10.1186/1741-7015-11-75)
Supplement: Additional file 1 — Registered systematic review protocol. On inception of this systematic review, the protocol was registered with the National Institute of Health Research International Prospective Register of Systematic Reviews, PROSPERO: CRD42011001459. [file 1741-7015-11-75-S1.PDF]

A systematic review of the maintenance effects of physical activity randomised controlled trials in healthy and ‘at risk’ older adults: LiveWell

Nicola Hobbs, Alan Godfrey, Falko Sniehotta, Lynn Rochester, Martin White, Linda Errington, John Mathers

Citation

Nicola Hobbs, Alan Godfrey, Falko Sniehotta, Lynn Rochester, Martin White, Linda Errington, John Mathers. A systematic review of the maintenance effects of physical activity randomised controlled trials in healthy and ‘at risk’ older adults: LiveWell. PROSPERO 2011:CRD42011001459 Available from
http://www.crd.york.ac.uk/PROSPERO/display_record.asp?ID=CRD42011001459

Review question(s)

Are physical activity interventions effective in changing physical activity behaviour?

What features of physical activity interventions are associated with intervention effectiveness in terms of changing behaviour?

What features of physical activity interventions are associated with intervention effectiveness in terms of maintenance of behaviour change?

Searches

The following databases were searched from January 2000 until December 2011:

Cochrane Central Register of Controlled Trials (CENTRAL) MEDLINE

EMBASE

PsycINFO

CINAHL

Web of Science

Scopus

ASSIA

Only records with an English language abstract will be assessed for eligibility.

Types of study to be included

Inclusion - randomised controlled trials.

Exclusion - non-randomised or quasi-randomised trials.

Condition or domain being studied

Physical activity behaviour.

Participants/ population

Inclusion - mean or median age of 55-70 years; healthy or at risk of disease i.e., hypertension, impaired glucose tolerance, overweight/obese, hyperlipidemia, dyslipidemia, family history of disease, metabolic syndrome, osteopenia etc.

Exclusion - recruited on the basis of a having a pre-existing chronic or acute medical condition; recruited on the basis of taking medication; institutionalised i.e., living in residential homes, nursing homes or being a hospital inpatient.

Intervention(s), exposure(s)

Inclusion - any interventions of any type of physical activity behaviour.

Exclusion - high performance training i.e., marathon runners and lab-based exercise intervention studies.

Comparator(s)/ control

Inclusion - Interventions could be compared with a no intervention control group, attention control (receiving attention

matched to length of intervention (e.g. general health check) and/or another intervention.

Exclude - interventions where the control or comparator intervention group did not measure physical activity.

Context

A study of a free-living, community-dwelling population, originating from a country of "high human development" (United Nations Human Development Index).

Outcome(s)

Primary outcomes

Objective or self-reported measure of physical activity behaviour.

Exclusion - physiological measures of physical activity i.e., energy expenditure, V02 max etc.

Studies with a follow up of at least 12 months after randomisation.

Secondary outcomes

Other behavioural outcomes related to lifestyle will be described and discussed but not included in data analyses i.e, diet, smoking, alcohol and social factors.

Studies with a follow up of at least 12 months after randomisation.

Data extraction, (selection and coding)

Study Selection

Eligible studies will be selected according to inclusion/exclusion criteria based on title and abstract. Two reviewers will independently assess all the articles. When it is unclear whether the study meets the inclusion criteria, the full text will be retrieved to clarify doubts. If there is disagreement between reviewers about studies to be included, a third reviewer will resolve discrepancies. Excluded studies and reasons for exclusion will be documented.

Data extraction

One reviewer will extract the data and enter it onto a data extraction form, another reviewer will check the data extracted. If there is any disagreement during this procedure, a third reviewer will resolve discrepancies. One reviewer will enter data into RevMan and another reviewer will independently verify it. No blinding procedures will be used for data pertaining author's names, journal or institutions. The information extracted from each study and presented in the 'characteristics of included studies' table will be described in the following structure:

1) Study design details

- Country

- Method of recruitment and sampling

- Units of randomization

- Flow diagram

- Follow up duration

- Appropriate analysis

2) Participants

- Type of population and setting

- Inclusion and exclusion criteria

- Baseline characteristics

- Recruitment rates

- Attrition rates at follow up

- Intention to treat analysis

3) Measurement description

- Type of measurement

- Evaluation points

4) Outcomes evaluated

- physical activity behaviours

- other health and social behaviours

5) Features of interventions

- Davidson (2003) criteria for reporting evidence based behavioral medicine

- behaviour change techniques

- theory

Risk of bias (quality) assessment

Two reviewers will independently assess methodological quality of the studies included before analysis. Methodological quality will be appraised using the Cochrane risk of bias tool covering the quality of random allocation concealment, description of withdrawals and dropouts, intention-to-treat-analysis, and blinding of participants, intervention providers

and outcome assessors. Each quality criterion will be assessed for risk of bias in the following way: 'low risk for bias', 'unclear risk for bias' and 'high risk for bias'. All data on quality gathered will be tabulated and accompanied by a description of quality for each study.

Strategy for data synthesis
A descriptive summary of all included studies will be provided. From our current knowledge of different types of outcomes (i.e. dichotomous and continuous), appropriate statistical techniques will be used for each (i.e. odd ratios and mean/standard mean difference, respectively).

We intend to conduct meta-analyses to calculate pooled effect sizes across studies.

Analysis of subgroups or subsets
Subgroup analyses based on the following characteristics are anticipated: study quality; length of follow up assessment; type of comparators; frequency of assessing physical activity behaviour; type and properties of physical activity behaviour assessment; direct vs, indirect feedback from physical activity assessment tool; behaviour change features (techniques, delivery and theory etc,).

Contact details for further information

Nicola Hobbs

Institute of Health and Society

Baddiley-Clark Building

Richardson Road

Newcastle University

Newcastle upon Tyne

NE2 4AX

nicki.hobbs@ncl.ac.uk

Organisational affiliation of the review
Newcastle University

www.ncl.ac.uk

Review team

Dr Nicola Hobbs, Newcastle University
Dr Alan Godfrey, Newcastle University
Dr Falko Sniehotta, Newcastle University
Professor Lynn Rochester, Newcastle University
Professor Martin White, Newcastle University
Ms Linda Errington, Newcastle University
Professor John Mathers, Newcastle University

Collaborators
Dr Vera Arujo-Soares, Newcastle University
Dr Sue Lord, Newcastle University

Anticipated or actual start date
01 November 2010

Anticipated completion date
26 December 2011

Funding sources/sponsors

LiveWell programme is funded through a Collaborative Grant from the Lifelong Health & Wellbeing initiative: grant ID number 91020

Conflicts of interest
None known

Language
English

Country
England

Subject index terms status
Subject indexing assigned by CRD

Subject index terms

Aged; Aged, 80 and over; Exercise; Humans; Randomized Controlled Trials as Topic

Date of registration in PROSPERO
02 August 2011

Date of publication of this revision
27 February 2013

**Stage of review at time of this submission**	**Started**	**Completed**
Preliminary searches	No	Yes
Piloting of the study selection process	No	Yes
Formal screening of search results against eligibility criteria	No	Yes
Data extraction	No	Yes
Risk of bias (quality) assessment	No	Yes
Data analysis	No	Yes
Prospective meta-analysis	No	Yes

PROSPERO
This information has been provided by the named contact for this review. CRD has accepted this information in good faith and registered the review in PROSPERO. CRD bears no responsibility or liability for the content of this registration record, any associated files or external websites.
