# Supplementary material for: Are behavioral interventions effective in increasing physical activity at 12 to 36 months in adults aged 55 to 70 years? a systematic review and meta-analysis
Source: BMC Med. 2013 Mar 19;11:75. doi: 10.1186/1741-7015-11-75 (PMC3681560; doi:10.1186/1741-7015-11-75)
Supplement: Additional file 2 — OVID Medline search strategy. Search terms and search strategy used to search for records in the OVID Medline electronic database. [file 1741-7015-11-75-S2.PDF]

OVID Medline search strategy <1950 to November Week 3 2010>

1 Motor Activity/ (62022)

2 physical$ activ$.ti,ab. (38174)

3 exp exercise/ (54186)

4 exercis$.ti,ab. (156620)

5 exp sports/ (89316)

6 dancing/ (1428)

7 gardening/ (275)

8 yoga/ (1071)

9 pilates.ti,ab. (48)

10 physical fitness/ (18227)

11 walk$.ti,ab. (50365)

12 exp Physical Exertion/ (50567)

13 or/1-12 (371556)

14 randomized controlled trial.pt. (307057)

15 exp animals/ not humans.sh. (3604852)

16 older$.ti,ab. (192022)

17 elder$.ti,ab. (141835)

18 senior$.ti,ab. (18365)

19 retir$.ti,ab. (10867)

20 Retirement/ (6756)

21 aged/ or middle aged/ (3282205)

22 ag?ing.ti,ab. (100659)

23 or/16-22 (3438910)

24 13 and 14 and 23 (12879)

25 24 not 15 (12876)

26 limit 25 to yr="2000 -Current" (7868)
