# Supplementary material for: Are behavioral interventions effective in increasing physical activity at 12 to 36 months in adults aged 55 to 70 years? a systematic review and meta-analysis
Source: BMC Med. 2013 Mar 19;11:75. doi: 10.1186/1741-7015-11-75 (PMC3681560; doi:10.1186/1741-7015-11-75)
Supplement: Additional file 3 — Table of characteristics of included trials in alphabetical order by author. Characteristics of the trials included in this review, including information on the study population, setting, physical activity outcome measures, assessment times, sample size in each intervention arm, content and delivery of intervention, and attrition rates. [file 1741-7015-11-75-S3.PDF]

Table of characteristics of included trials in alphabetical order by author

**Trial Name/Publications/ Location/Design**	**Participants/Setting**	**Physical Activity Outcomes/Assessment Time**	**Intervention Details**
Patient-motivated Health Promotion Plan (PHPP)<br>*Babazono 2007<br>Japan<br>RCT	*Description of participants*: Members of the National Health Insurance, Umi Town<br>*Inclusion criteria:* BP Systolic 130-159, Diastolic 85-99 mmHg OR HbA1c level ≥5.6%<br>*Exclusion criteria:* Physicians judgement<br>*Gender:* 54% female<br>*Mean age (SD):* 64.4 years (7.5)	**Steps/day (pedometer)**<br>Baseline & **12 months**	**Intervention condition**<br>*Sample size:* 50<br>*Delivery:* Health professional; face-to-face individual; healthcare premises and at home<br>*Content:* To establish a healthy lifestyle by setting and prioritising goals and selecting lifestyle improvements. Active intervention period was ≤1 month with 1 intervention contact<br>*Attrition*: 4% at 12 months<br>**Control condition**<br>*Sample size:* 49<br>*Content*: Leaflets on how to enhance exercise<br>*Attrition*: 16% at 12 months
Green prescription intervention<br>***Elley 2003**; Elley 2004; Elley 2005<br>New Zealand (& Australia)<br>Clustered RCT	*Description of participants*: Patients attending rural & urban GP in central and western region of Waikato, with less than 30min/day/wk moderate to vigorous PA<br>*Inclusion criteria:* Aged 40-79 years. Answering no to: "As a rule, do you do at least half an hour of moderate or vigorous exercise (such as walking or a sport) on five or more days of the week?"	**PA duration (Kcal/kg/week total)**<br>PA duration (Kcal/kg/week leisure time)<br>(both computed using a self-administered questionnaire from the Auckland heart study, which prompts for recall of physical activity over three months, to estimate total and leisure time	**Intervention condition**<br>*Sample size:* 451<br>*Delivery:* Health professional; face-to-face individual/telephone/printed material; healthcare premises<br>*Content*: Motivational interviewing techniques to advice on PA, give green prescription and provide feedback. Active intervention period was ≤1 month with 1 intervention contact<br>*Attrition*: 15% at 12 months<br>**Control condition**

Trial Name/Publications/ Location/Design	Participants/Setting	Physical Activity Outcomes/Assessment Time	Intervention Details
	Exclusion criteria: Patients were excluded if practice personnel considered them to be too unwell to participate, if they did not understand English, or if they were expecting to leave the region.<br>*Gender:* 50.5% Female<br>*Mean age (SD):* 57.9 (11.2)	expenditure of energy)<br>PA duration (mins/week leisure exercise)<br>(computed using a 3-month PA recall questionnaire and PA diary)<br>Baseline & **12 months**	*Sample size:* 427<br>*Content*: Received usual care – received intervention at the end of the study.<br>*Attrition*: 15% at 12 months
Halbert 2000<br>Australia<br>RCT	*Description of participants*: Sedentary adults based in the community<br>*Inclusion criteria:* Sedentary adults aged 60+ years<br>*Exclusion criteria*: Having a cerebrovascular or ischemic cardiac event in the previous 6 months, etc.<br>*Gender:* 54% Female<br>*Mean age (SD):* 67.6 (6.7)	**PA duration (mins/session vigorous exercise)**<br>**PA duration (mins/session walking)**<br>PA duration (session/wk vigorous exercise)<br>PA duration (session/wk walking)<br>Baseline, 3, 6 & **12 months**	**Intervention condition**<br>*Sample size:* 149<br>*Delivery:* Unclear provider; face-to-face individual/printed material; healthcare premises<br>*Content*: Individualised advice about the benefits of PA and a pamphlet containing a plan for PA for the next three months through organised sessions. Active intervention period was 6 months with 5 intervention contacts<br>*Attrition*: 17% at 12 months<br>**Control condition**<br>*Sample size:* 150<br>*Content*: Pamphlet promoting good nutrition for older adults, which was discussed for 20 minutes<br>*Attrition*: 6% at 12 months
Hartslag (Heartbeat) Limburg project<br>Harting 2006	*Description of participants*: Patients of GP practice's stratified by socioeconomic class and preventive	**PA duration (% meeting PA target)**<br>(short validated questionnaire	**Intervention condition**<br>*Sample size:* 670<br>*Delivery:* Health professional; face-to-face

Trial Name/Publications/ Location/Design	Participants/Setting	Physical Activity Outcomes/Assessment Time	Intervention Details
The Netherlands<br>Clustered RCT	orientation<br>*Inclusion criteria:* Participants had to have a greater than 20% risk of incurring a cardiovascular event within 10 years<br>*Exclusion criteria*: Not reported<br>*Gender:* 33.8% Female<br>*Mean age (SD):* 62.3 (9.1)	addressing 11 activity categories scored in minutes per day and days per week, producing an indirect PA score. In addition, patients were asked to indicate the number of days per week on which they were usually physically active for more than 30 min (direct assessment). Patients who met the norm of being sufficiently physically active, based on direct plus indirect assessments, were classified as meeting the recommended target.<br>Baseline, 4 & **18 months**	individual/telephone; unspecified setting<br>*Content:* Cardiovascular risk profile and theory-based stage-matched health counselling for healthy lifestyle change. Active intervention period was 2 months with 2 intervention contacts<br>*Attrition*: 16% at 4 months, 24% at 18 months<br>**Control condition**<br>*Sample size*: 630<br>*Content*: Usual care from GP<br>*Attrition*: 8% at 4 months, 15% at 18 months
The Sex Hormones And Physical Exercise (SHAPE) trial<br>Hertogh 2010; Monninkhof 2007<br>The Netherlands<br>RCT	*Description of participants*: Low active postmenopausal women<br>*Inclusion criteria:* Sedentary postmenopausal women<br>*Exclusion criteria*: 12 months since last menses, use of hormone replacement or oral contraceptives in past 6 months, active life style, BMI > 40 or < 22,	**PA duration (MET hours/week)**<br>PA duration (total Baecke score)<br>PA duration (sport score)<br>PA duration (household score)<br>PA duration (leisure score)	**Intervention condition**<br>*Sample size*: 96<br>*Delivery:* Health professional; face-to-face group/telephone; healthcare premises<br>*Content*: Group combined endurance and strength training programme over a period of 12 months. Active intervention period was 12 months with 40 intervention contacts.

Trial Name/Publications/ Location/Design	Participants/Setting	Physical Activity Outcomes/Assessment Time	Intervention Details
	currently on or planning to go on a strict diet, ever diagnosed with breast cancer, diagnosis of other types of cancer in the past 5 years, diabetes mellitus or other endocrine related diseases, disorders or diseases that might impede the participation in the exercise programme, alcohol or drug abuse, maintenance use of corticosteroids, use of beta blockers & smoking<br>*Gender:* 100% Female<br>Mean age (SD): 58.7 (4.4)	(computed using Modified Baecke questionnaire to assess habitual PA in elderly - sport, household and leisure are subscales; METS calculated based on sport and leisure score)<br>Baseline, **12 & 24 months**	*Social support component:* None<br>*Attrition*: 2% at 12 months, 19% at 24 months<br>**Control condition**<br>*Sample size:* 93<br>*Content*: Requested to retain their habitual exercise pattern. They also receive newsletters<br>*Attrition*: 6% at 12 months, 24% at 24 months
King 2000<br>USA<br>RCT	*Description of participants*: Residents of Sunnyvale, California<br>*Inclusion criteria:* Current resident of Sunnyvale; 65+ years; absence of cardiovascular disease or stroke; regularly active no more than twice a week during the preceding 6 months; free of musculoskeletal problems that would prevent moderate levels of PA; stable on all medications during the previous 6-month period; and willingness to accept randomisation<br>*Exclusion criteria*: Telephone interview (medical exclusions; already	**PA duration (cal/kg/day MVPA)**<br>(computed using Community Healthy Activities Model Program for Seniors instrument to assess self-reported PA in elders and converted into energy expenditure)<br>Baseline & **12 months**	**Intervention condition (fit & firm)**<br>*Sample size:* 50<br>*Delivery:* Health professional; face-to-face group/telephone; community setting<br>*Content*: Encouraged to participate in two exercise classes/week and to exercise at home at least twice a week. Active intervention period was 12 months with 228 intervention contacts.<br>*Attrition*: Unclear<br>**Intervention condition (stretch & flex)**<br>*Sample size:* 46<br>*Delivery:* Health professional; face-to-face group/telephone; community setting<br>*Content*: Encouraged to participate in two exercise

Trial Name/Publications/ Location/Design	Participants/Setting	Physical Activity Outcomes/Assessment Time	Intervention Details
	physically active based on study eligibility criteria; some had plans to move from the area within the next 2 years);<br>*Gender:* 61.2% Female<br>*Mean age (SD):* 70 (3.9)		classes each week and to exercise on their own at home at least twice a week. Active intervention period was 12 months with 16 intervention contacts.<br>*Attrition*: Unclear
Community Health Advice by Telephone (CHAT) trial<br>***King 2007**; King 2002<br>USA<br>RCT	*Description of participants*: Older adults in the community<br>*Inclusion criteria:* 55+ years; not initially engaged in more than 60 minutes per week of moderate-intensity or vigorous PA over the previous 6 months; free of any medical condition that would limit participation in moderate-intensity exercise; BMI ≤40; average alcohol intake ≤3 drinks per day; able to speak and understand English; regular access to a touch tone phone; not planning to move from the area over the study period; willing to be randomised<br>*Exclusion criteria*: As above<br>*Gender:* 69.3% Female<br>*Mean age (SD):* 60.8 (5.5)	**PA duration (mins/week MVPA)**<br>PA duration (Kcal/kg/day MVPA)<br>(computed using Stanford 7-day PA recall questionnaire)<br>PA duration (Kcal/kg/day MVPA)<br>PA duration (mins/week MVPA)<br>(computed using Community Healthy Activities Model Program for Seniors)<br>Baseline, 6 & **12 months**	***Intervention condition (human advice intervention)**<br>*Sample size:* 73<br>*Delivery:* Health professional; face-to-face individual/telephone; home<br>*Content*: Telephone-assisted PA counselling by a trained health educator.<br>Active intervention period was 12 months with 16 intervention contacts.<br>*Attrition*: 10% at 12 months<br>**Intervention condition (automated advice intervention)**<br>*Sample size:* 75<br>*Delivery:* Health professional; face-to-face individual/telephone; home<br>*Content*: Telephone-assisted PA counselling by an automated telephone-linked computer system.<br>Active intervention period was 12 months with 16 intervention contacts.<br>*Attrition*: 19% at 12 months

Trial Name/Publications/ Location/Design	Participants/Setting	Physical Activity Outcomes/Assessment Time	Intervention Details
			**Control condition**<br>*Sample size:* 70<br>*Content*: Offered weekly health education classes that focused on a variety of non-PA topics of interest to middle and older aged adults such as nutrition and home safety, and were asked not to change their usual PA patterns<br>*Attrition*: 11% at 12 months
The Women On the Move through Activity And Nutrition (WOMAN) trial<br>***Kuller 2006**;<br>Newman 2009<br>USA<br>RCT	*Description of participants*: Postmenopausal women on hormone therapy (HT)<br>*Inclusion criteria:* 52 – 62 years with at least 2 years HT, waist circumference (WC) ≥ 80cm, LDL-C between 100 & 160, BMI 25 – 39.9, BP < 160/95 at screening but 140/90 at randomisation, a Beck Depression Index of <20, no diagnosis of psychotic, anxiety, no eating disorder, not depressed, non-diabetic, no use of cholesterol lowering medication, no history of cancer in 2 years, ability to walk 400m with HR >40 & <135 beats throughout, willing to be randomised<br>*Exclusion criteria*: Not reported<br>*Gender:* 100% Female	**Steps/day (pedometer)**<br>PA duration (MET hours/week) (computed using Modifiable Activity Questionnaire)<br>Baseline & **18 months**	**Intervention condition**<br>*Sample size:* 222<br>*Delivery:* Health professional; face-to-face group; unspecified setting<br>*Content*: Lifestyle changes promoting 150 mins/week of moderate intensity PA, reduction in calorific intake and improved diet. Active intervention period was 12 months with 40 intervention contacts.<br>*Attrition*: 31% at 18 months<br>**Control condition**<br>*Sample size:* 194<br>*Content*: Health education seminars from Dietary Guidelines for America<br>*Attrition*: 15% at 18 months

**Trial Name/Publications/ Location/Design**	**Participants/Setting**	**Physical Activity Outcomes/Assessment Time**	**Intervention Details**
	Mean age (SD): 56.0 (Not reported)		
Diabetes Prevention Study (DPS)<br>***Lindstrom 2010;**<br>Lindstrom 2003;<br>Lindstrom 2003(b);<br>Tuomilehto 2001<br>Finland<br>RCT	*Description of participants*: Overweight subjects & relatives of those with type 2 diabetes (DPS)<br>*Inclusion criteria:* 40–64 years with BMI >25 kg/m2 at screening and the mean value of two 75-g OGTTs in the impaired glucose tolerance range based on WHO criteria<br>*Exclusion criteria*: Diagnosis of diabetes mellitus or the presence of a chronic disease, etc.<br>*Gender*: 67% Female<br>*Mean age (SD):* 55.0 (7.0)	**PA duration (% meeting PA target of >4 hours exercise per week**<br>(computed by a shift to a higher category of frequency and intensity of PA)<br>**PA duration (mins/week total PA)**<br>PA duration **(**mins/week MVPA)<br>(computed using Leisure Time PA questionnaire; Kuopio Ischemic Heart Disease Risk Factor)<br>Baseline, **12 & 36 months**	**Intervention condition**<br>*Sample size:* 265<br>*Delivery:* Health professional; face-to-face individual/group/telephone/printed material; healthcare premises<br>*Content*: Individual lifestyle counselling to increase PA and individually tailored circuit-type training. Active intervention period was 12 months with 7 intervention contacts<br>*Attrition*: 3% at 12 months; 4% at 24 months<br>**Control condition**<br>*Sample size*: 257<br>*Content:* Educational: General information (verbal & written) on lifestyle and diabetes risk. Advised on how to reduce BMI, alcohol and to stop smoking<br>*Attrition:* 2% at 12 months; 4% at 24 months
Keep Active Minnesota (KAM Study)<br>***Martinson** 2010;<br>Crain 2010;<br>Sherwood 2008<br>USA<br>RCT	*Description of participants*: 50 – 70 year olds from one large managed care organisation<br>*Inclusion criteria:* Self-reported that they had increased their physical activity over the last year & accumulating an average 30m moderate to vigorous PA at least 2 days per week	**PA duration (Kcal/wk total PA)**<br>PA duration (Kcal/wk MVPA)<br>(computed using Community Healthy Activities Model Program for Seniors instrument to assess self-	**Intervention condition**<br>*Sample size:* 523<br>*Delivery:* Health professional; face-to-face group/telephone/printed material; home<br>*Content*: Interactive telephone and mail-based PA support program including coaching, assignments, goal setting and self-monitoring (pedometer). Active intervention period was 4 months with 7 intervention

Trial Name/Publications/ Location/Design	Participants/Setting	Physical Activity Outcomes/Assessment Time	Intervention Details
	over the past 4 weeks. Enrolled in a health plan.<br>*Exclusion criteria*: Excluded if they had a modified Charlson co-morbidity score >3, or had diagnosis of various diseases<br>*Gender:* 72% Female<br>*Mean age (SD):* 57.1 (5.1)	reported frequency and duration of a range of common activities and converted into weekly kcal expenditure)<br>Baseline, 6, **12 & 24 months**	contacts<br>*Attrition*: 2% at 6 months; 0% at 12 months; 10% at 24 months<br>**Control condition**<br>*Sample size:* 526<br>*Content*: Usual care, information leaflet and newsletters<br>*Attrition*: 2% at 6 months; 1% at 12 months; 19% at 24 months
*Lawton 2008<br>New Zealand<br>RCT	*Description of participants*: Sedentary women recruited from 2 sources: cohort (50-74yrs) of postmenopausal women from 10 primary care practices & the rest 50-70yrs (40-60yrs for Maori & Pacific women) recruited from 13 primary care practices including 2 Maori health clinics<br>*Inclusion criteria:* Aged 40-74 physically inactive, as determined by one question screening tool: "As a rule, do you do at least half an hour of moderate or vigorous exercise (such as walking or a sport) on five or more days of the week?"<br>*Exclusion criteria*: medical condition that might be adversely affected by	**PA duration (mins PA/week)**<br>**PA duration (% completing at least 150 mins PA/week)**<br>(both computed using the long form of authors' PA questionnaire)<br>Baseline, **12 & 24 months**	**Intervention condition**<br>*Sample size:* 544<br>*Delivery:* Health professional; face-to-face individual/telephone; healthcare premises<br>*Content*: Brief counselling using motivational interviewing techniques to increase PA. The exercise advice is written on a "green script," which is given to the patient and faxed to a community based exercise facilitator who provides telephone support. Active intervention period was 9 months with 7 intervention contacts.<br>*Attrition*: 8% at 12 months, 3% at 24 months<br>**Control condition**<br>*Sample size:* 545<br>*Content*: Received usual care<br>*Attrition*: 7% at 12 months, 4% at 24 months

**Trial Name/Publications/ Location/Design**	**Participants/Setting**	**Physical Activity Outcomes/Assessment Time**	**Intervention Details**
	increasing their PA, as determined by the PA readiness questionnaire & subsequent assessment by GP<br>*Gender:* 100% Female<br>*Mean age (SD):* 58.9 (7.0)		
*McTiernan 2007<br>USA<br>RCT	*Description of participants*: Sedentary/unfit patients from gastroenterology practices<br>*Inclusion criteria:* 40 – 75 years, <90 min/wk of moderate-to-vigorous intensity over the previous 3 months, <2 alcohol drinks/d, had no personal history of invasive cancer or other disease & had normal complete blood count & blood chemistries.<br>*Exclusion criteria*: Unwillingness to be randomised, non-sedentary and insufficient time availability<br>*Gender:* 49.5% Female<br>*Mean age (SD):* 55.2 (6.8)	**Steps/day (pedometer)**<br>**PA duration (mins/week MVPA)** (computed using Minnesota Leisure Time PA questionnaire)<br>Baseline, 3, 6, 9 & **12 months**	**Intervention condition**<br>*Sample size:* 100<br>*Delivery:* Health professional; face-to-face (unclear if individual or group); healthcare premises and home<br>*Content*: Facility and home-based exercise programme with regular monitoring and feedback. Active intervention period was 12 months with 156 potential intervention contacts<br>*Attrition*: 2% at 3 months, 1% at 12 months<br>**Control condition**<br>*Sample size:* 102<br>*Content*: Asked not to change diet or PA patterns<br>*Attrition*: 3% at 3 months, 0% at 12 months
***Opdenacker 2008**; Opdenacker 2009<br>Belgium<br>RCT	*Description of participants*: Retired university employees & members of socio-cultural organisations for seniors<br>*Inclusion criteria:* >60 years, not having participated systematically in any endurance or strength training in the 2	**Vector magnitude/week (accelerometer)**<br>**Steps/day (pedometer)**<br>**PA duration (kcal/week leisure PA)**<br>PA duration (kcal/week	***Intervention condition (lifestyle intervention)**<br>*Sample size:* 60<br>*Delivery:* Health professional; face-to-face individual/group/telephone; home<br>*Content*: Home-based lifestyle programme to integrate PA into daily routines with pedometer.

Trial Name/Publications/ Location/Design	Participants/Setting	Physical Activity Outcomes/Assessment Time	Intervention Details
	year period preceding the study. Not being physically active at moderate intensity for more than 2h a week at recruitment<br>*Exclusion criteria*: All pathologies with contraindications for involvement in a training program such as stroke, etc. and all neurodegenerative diseases (PD, etc.) Intake of medication known to affect exercise capacity or bone density<br>*Gender:* 47.7% Females<br>*Mean age (SD):* 67.1 (4.5)	active transportation<br>PA duration (kcal/week household/garden activities)<br>Baseline, **12 & 24 months**	Active intervention period was 12 months with 23 intervention contacts<br>*Attrition*: 8% at 12 months, 23% at 24 months<br>***Intervention condition (structured intervention)**<br>*Sample size:* 60<br>*Delivery:* Health professional; face-to-face individual/group/telephone; home<br>*Content*: Three weekly supervised sessions of 60–90 min in a fitness centre Participants exercised in groups of 10 and were supervised by the two instructors.<br>Active intervention period was 12 months with 8 intervention contacts<br>*Attrition*: 3% at 12 months, 18% at 24 months<br>**Control condition**<br>*Sample size:* 60<br>*Content*: Participated in the measurements only with no feedback<br>*Attrition*: 5% at 12 months, 32% at 24 months
The Step Test Exercise Prescription Stage (STEPS) trial<br>Petrella 2010<br>Canada<br>RCT	*Description of participants*: Patients receiving primary health care from family medicine clinics (urban & rural)<br>*Inclusion criteria:* 55 – 85 years, inactive lifestyle; readiness to increase their PA levels based on their stage of change; able to read and write English;	**PA duration (Kcal/kg/day during light PA**<br>(computed using PA recall questionnaire)<br>Baseline & **12 months**	**Intervention condition**<br>*Sample size:* 193<br>*Delivery:* Health professional; face-to-face individual/telephone; healthcare premises<br>*Content*: Individualized exercise prescriptions based on sub-maximal step test results, and counselling and support based on their stages of exercise behaviour.

Trial Name/Publications/ Location/Design	Participants/Setting	Physical Activity Outcomes/Assessment Time	Intervention Details
	the absence of exclusionary medical conditions, severe systemic or musculoskeletal disease preventing increased PA, or major psychiatric disease<br>*Exclusion criteria*: Not reported<br>*Gender:* 57.2% Female<br>*Mean age (SD):* 64.9 (7.1)		Active intervention period was 12 months with 16 intervention contacts.<br>*Attrition*: 12% at 12 months<br>**Control condition**<br>*Sample size:* 167<br>*Content*: Individualized exercise prescriptions based on sub-maximal step test results<br>*Attrition*: 4% at 12 months
*Racette 2008;<br>Racette 2006<br>USA<br>RCT	*Description of participants*: Healthy adults recruited from the St. Louis metropolitan area<br>*Inclusion criteria:* Healthy adults between 50 and 60 years of age with a BMI between 23.5 and 29.9, non-smokers, not exercising regularly; all women postmenopausal<br>*Exclusion criteria*: Any medical conditions, smokers<br>*Gender:* 64.5% Female<br>*Mean age (SD):* 57 (1.0)	**PA duration (MET hours/day above rest)** (computed using Stanford 7-day PA recall questionnaire)<br>Baseline,1, 3, 6, 9 & **12 months**	***Intervention condition (exercise)**<br>*Sample size:* 19<br>*Delivery:* Health professional; face-to-face individual and/or group; unspecified setting<br>*Content*: Instructed to increase daily PA energy expenditure without changing their dietary intake. Active intervention period was 12 months with 104 intervention contacts.<br>*Attrition*: Not reported<br>**Intervention condition (diet)**<br>*Sample size:* 19<br>*Delivery:* Health professional; face-to-face individual and/or group; unspecified setting<br>*Content*: Instructed to modify their daily energy intake without changing their PA patterns. Active intervention period was 12 months with 104 intervention contacts.<br>*Attrition*: Not reported

Trial Name/Publications/ Location/Design	Participants/Setting	Physical Activity Outcomes/Assessment Time	Intervention Details
			**Control condition**<br>*Sample size:* 10<br>*Content*: Participants did not receive a diet or exercise prescription<br>*Attrition*: Not reported
The Massachusetts WISEWOMAN Project<br>*Stoddard 2004<br>USA<br>Clustered RCT	*Description of participants*: Uninsured and underinsured women receiving CVD screening (Massachusetts WISEWOMAN project, MWWP). Part of the Massachusetts Breast & Cervical Cancer Initiative (MBCCI)<br>*Inclusion criteria:* Aged ≥50 years, uninsured or underinsured, eligible for MBCCI enrolment<br>*Exclusion criteria*: Not reported<br>*Gender:* 100% Female<br>*Mean age (SD):* 58 (Not reported)	**PA duration (% doing adequate PA - at least 30 mins of MVPA at least 3 times per week)** (computed using two self-report questions)<br>Baseline & **12 months**	**Intervention condition**<br>*Sample size:* 600<br>*Delivery:* Health professional; face-to-face individual/group/printed material; healthcare premises<br>*Content*: Enhanced lifestyle intervention focusing primarily on nutrition & PA to reduce CVD risk. Length of active intervention period and number of intervention contacts was not specified.<br>*Attrition*: 20% at 12 months<br>**Control condition**<br>*Sample size:* 475<br>*Content*: All participants received a set of low-literacy fact sheets on cholesterol, blood pressure, blood glucose, nutrition, PA & stress reduction<br>*Attrition*: 27% at 12 months
Vitalum trial<br>* **van Keulen** 2011; van Keulen 2008<br>The Netherlands<br>RCT	*Description of participants*: From 23 Dutch general practices<br>*Inclusion criteria:* 45 – 70 years; about 50% diagnosed by their GP as hypertensive according to the	**PA duration (hours/week)** (computed using 28-item modified Community Healthy Activities Model Program for Seniors)	***Intervention condition (combined tailored print & telephone motivational monitoring)**<br>*Sample size:* 408<br>*Delivery:* Participant under instruction; printed material/telephone; home

Trial Name/Publications/ Location/Design	Participants/Setting	Physical Activity Outcomes/Assessment Time	Intervention Details
	International Classification of Primary Care, about 50% male; not participating in other studies according to the GP database; & maximum one person per address<br>*Exclusion criteria*: Failing to meet at least 2 public health guidelines: PA and either fruit or vegetable consumption. Intellectual disability, unable to speak/read Dutch, suffering from some disorder<br>*Gender:* 45% Female<br>*Mean age (SD):* 57.2 (7.1)	Baseline, 6, **12 & 18 months**	*Content*: Two tailored print letters and two telephone motivational interviews. Given a pedometer. Active intervention period was 10 months with 5 intervention contacts.<br>*Attrition*: 30% at 18 months<br>**Intervention condition (tailored print communication only)**<br>*Sample size:* 405<br>*Delivery:* Participant under instruction; printed material; home<br>*Content*: Four tailored print letters and given a pedometer. Active intervention period was 10 months with 5 intervention contacts.<br>*Attrition*: 33% at 18 months<br>**Intervention condition (telephone motivational interviewing only)**<br>*Sample size:* 407<br>*Delivery:* Participant under instruction; telephone; home<br>*Content*: Four telephone motivational interviews and given a pedometer. Active intervention period was 10 months with 5 intervention contacts.<br>*Attrition*: 26% at 18 months<br>**Control condition**<br>*Sample size:* 409<br>*Content*: Received one tailored letter based on the

**Trial Name/Publications/ Location/Design**	**Participants/Setting**	**Physical Activity Outcomes/Assessment Time**	**Intervention Details**
			last follow-up questionnaire.<br>*Attrition*: 20% at 18 months
*van Stralen 2010<br>the Netherlands<br>Clustered RCT	*Description of participants*: Older adults from 6 Dutch Regional Municipal Health Councils<br>*Inclusion criteria:* Aged 50+ years<br>*Exclusion criteria*: Not reported<br>*Gender:* 57% Female<br>*Mean age (SD):* 64 (8.6)	**PA duration (mins/week total PA)**<br>(computed using the Short Questionnaire to Assess Health-enhancing PA – total mins per activity calculated by multiplying frequency of activity (days/week) by duration of activity regardless of intensity)<br>Baseline & **12 months**	***Intervention condition (environmentally tailored)**<br>*Sample size:* 737<br>*Delivery:* Participant under instruction; printed material; home<br>*Content*: Three tailored letters including personalised PA advice and tailored information about PA opportunities in specific environment. Active intervention period was 4 months with 5 intervention contacts.<br>*Attrition*: 35% at 6 months, 38% at 12 months<br>**Intervention condition (basic)**<br>*Sample size:* 654<br>*Delivery:* Participant under instruction; printed material; home<br>*Content*: Three tailored letters including personalised PA advice. Active intervention period was 4 months with 5 intervention contacts.<br>*Attrition*: 33% at 6 months, 34% at 12 months<br>**Control condition**<br>*Sample size:* 586<br>*Content*: Waiting list control participants received nothing during the intervention period<br>*Attrition*: 17% at 6 months, 21% at 12 months

Trial Name/Publications/ Location/Design	Participants/Setting	Physical Activity Outcomes/Assessment Time	Intervention Details
*Wang 2008<br>USA<br>RCT	*Description of participants*: Women recruited from the Piedmont Triad area of North Carolina, USA<br>*Inclusion criteria:* 50–70 years, & at least one year without menses, overweight or obese (BMI = 25–40 & waist girth > 88 cm), non-smoking, postmenopausal, not on hormone therapy, sedentary (<15 min of exercise, 2 times/wk) in the past 6 months & weight-stable (< 5% weight change) for at least 6 months prior to enrolment.<br>*Exclusion criteria*: Participants with evidence of untreated hypertension, hypertriglyceridemia (triglycerides > 400 mg/dl), insulin-dependent diabetes, active cancer, liver, renal or haematological disease, or other medical disorders.<br>*Gender:* 100% Female<br>*Mean age (SD):* 58.6 (5.2)	**PA duration (PA/week)** (computed using Physical Activity Scale for the Elderly)<br>kJ/day PA energy expenditure (accelerometer)<br>Baseline, 6 & **12 months**	**Intervention condition (diet & high exercise)**<br>*Sample size:* 13<br>*Delivery:* Unspecified provider; face-to-face (unclear if individual or group); university facility<br>*Content*: Exercise prescription of centre-based treadmill walking three days/week at a target heart rate progressing to 70-75% of maximum, meal provision and food diaries. Active intervention period was 5 months with 60 intervention contacts.<br>*Attrition*: 15% at 12 months<br>**Intervention condition (diet & low exercise)**<br>*Sample size:* 13<br>*Delivery:* Unspecified provider; face-to-face (unclear if individual or group); university facility<br>*Content*: Exercise prescription of centre-based treadmill walking three days/week at a target heart rate of 45-50% of maximum, meal provision and food diaries. Active intervention period was 5 months with 60 intervention contacts.<br>*Attrition*: 8% at 12 months<br>**Control condition**<br>*Sample size:* 15<br>*Content*: Diet only group<br>*Attrition*: 27% at 12 months
Wageningen Approach against fat Accumulation and	*Description of participants*: Recent **retirees** recruited from pre-retirement workshops as offered by employers to	**PA duration (hours/day total PA)**<br>PA duration (min/week –	**Intervention condition**<br>*Sample size:* 209<br>*Delivery:* Unspecified provider; internet/printed;

**Trial Name/Publications/ Location/Design**	**Participants/Setting**	**Physical Activity Outcomes/Assessment Time**	**Intervention Details**
weight Gain (WAAG) trial<br>***Werkman** 2010; Werkman 2006<br>The Netherlands<br>Clustered RCT	10% of Dutch population<br>*Inclusion criteria:* 55 – 65 years, recently retired, apparently healthy and not undergoing any medical treatment that might affect the outcome measures<br>*Exclusion criteria*: Not reported<br>*Gender:* 14.8% Female<br>*Mean age (SD):* 59.5 (2.4)	daily routine PA)<br>PA duration (min/week recreational sports PA)<br>PA duration (sum of household activities)<br>(all measures computed using the Dutch version of the Physical Activity Scale for the Elderly).<br>Baseline, **12 & 24 months**	home<br>*Content*: Printed and computer tailored modules to improve PA and diet. Active intervention period was 12 months with 15 intervention contacts<br>*Attrition*: 2% at 12 months, 11% at 24 months<br>**Control condition**<br>*Sample size:* 204<br>*Content*: Newsletters and general information about the study.<br>*Attrition*: 5% at 12 months, 9% at 24 months
Pre-diabetes Risk Education and Physical Activity Recommendations & Encouragement (PREPARE)<br>***Yates 2009;** Yates 2010; Yates 2008<br>UK<br>RCT	*Description of participants*: Overweight & obese individuals with impaired glucose intolerance (IGT) at University Hospitals of Leicester<br>*Inclusion criteria:* Overweight/obese BMI ≥25 or ≥23 for South Asians<br>*Exclusion criteria*: Taking steroids & diagnosis of Type 2 diabetes<br>*Gender:* 45% Female<br>*Mean age (SD):* 64 (9)	**Steps/day (pedometer)**<br>**PA duration (MET min/week total MVPA)**<br>**PA duration (MET min/week walking)**<br>(METs computed using long version of last 7-days form of International Physical Activity Questionnaire)<br>Baseline, 3, 6 & **12 months**	***Intervention condition (PREPARE plus pedometer)**<br>*Sample size:* 35<br>*Delivery:* Health professional; face-to-face group; healthcare premises<br>*Content*: Education programme addressing the causes, complications, timeline, and identity of impaired glucose tolerance; and addressing the perceived effectiveness of PA as a treatment. Participants provided with a pedometer and encouraged to set personalised steps-per-day goals based on their baseline ambulatory activity level. Active intervention period was 1 month with 1 intervention contact<br>*Attrition*: 6% at 3 months , 9% at 6 months, 13% at 12 months

Trial Name/Publications/ Location/Design	Participants/Setting	Physical Activity Outcomes/Assessment Time	Intervention Details
			**Intervention condition (PREPARE only)**<br>*Sample size:* 33<br>*Delivery:* Health professional; face-to-face group; healthcare premises<br>*Content*: Education programme addressing the causes, complications, timeline, and identity of impaired glucose tolerance; and addressing the perceived effectiveness of PA as a treatment. Participants encouraged to set time-based goals to match advice give to pedometer group. Active intervention period was 1 month with 1 intervention contact.<br>*Attrition*: 12% at 3 months , 3% at 6 months, 4% at 12 months<br>**Control condition**<br>*Sample size:* 35<br>*Content*: Brief information sheet in the mail, detailing the likely causes, consequences, etc. associated with impaired glucose tolerance, along with information about how PA can be used to treat/control the condition<br>*Attrition*: 9% at 3 months , 9% at 6 months, 17% at 12 months

MVPA = moderate to vigorous physical activity; PA = physical activity.

An asterisk before the author name (*NAME) indicates that the trial was included in meta-analysis; an asterisk before the intervention condition (*INTERVENTION CONDITION) denotes which intervention arm was the most intensive and used in analyses to compare with the control arm; for trials reported in multiple publications, the reference in **bold** indicates the publication used to denote the trial in the review.

Physical activity outcomes and assessment times in **bold** indicate the data used in this review
