# Supplementary material for: Are behavioral interventions effective in increasing physical activity at 12 to 36 months in adults aged 55 to 70 years? a systematic review and meta-analysis
Source: BMC Med. 2013 Mar 19;11:75. doi: 10.1186/1741-7015-11-75 (PMC3681560; doi:10.1186/1741-7015-11-75)

Risk of bias figure: judgements about each risk of bias item presented as percentages across 21 included studies

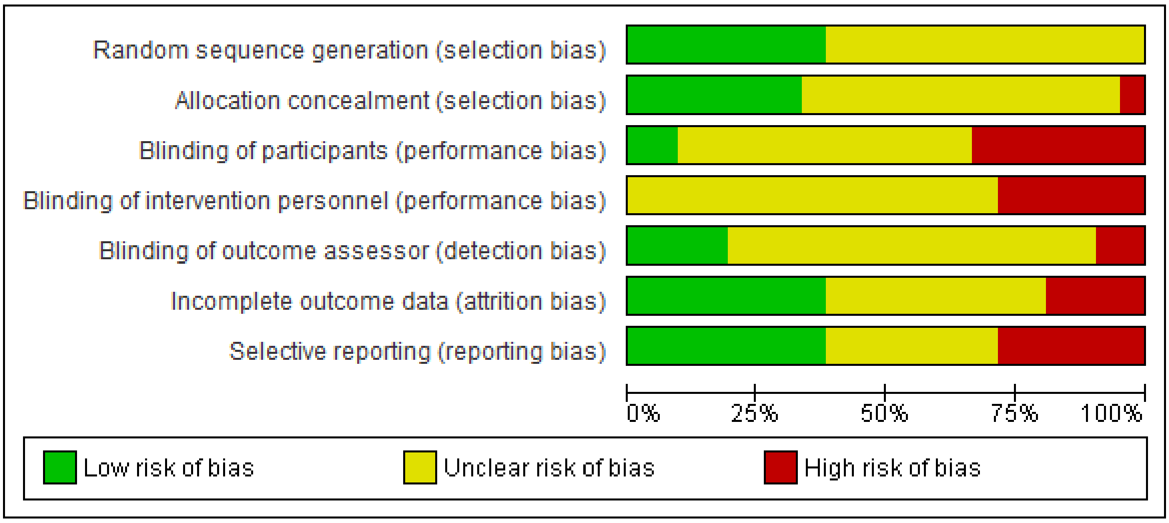

Supplement: Additional file 5 — Cochrane risk of bias figure. Risk of bias present in the trials included in this review based on the Cochrane risk of bias tool. Judgements about each risk of bias item are presented as percentages across the 21 included trials. The green bars represent the percentage of trials rated as high quality (low risk of bias) on each item, the yellow bars represent the percentage of trials where judgements could not be made (unclear risk of bias) on each item, and the red bars represent the percentage of trials rated as poor quality (high risk of bias) on each item. [file 1741-7015-11-75-S5.PDF]
