# Supplementary material for: Are behavioral interventions effective in increasing physical activity at 12 to 36 months in adults aged 55 to 70 years? a systematic review and meta-analysis
Source: BMC Med. 2013 Mar 19;11:75. doi: 10.1186/1741-7015-11-75 (PMC3681560; doi:10.1186/1741-7015-11-75)

Funnel plot of trials reporting pedometer step-counts (steps/day) at 12 months

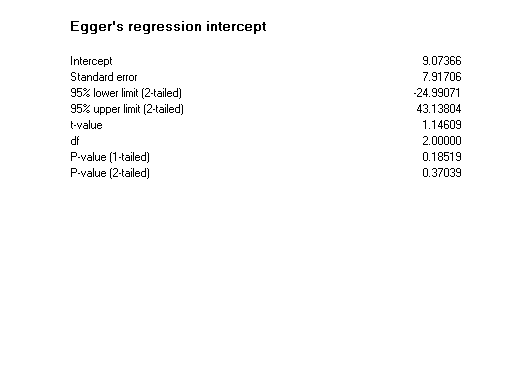


Funnel plot of trials reporting duration of PA at 12 months – continuous outcome measures

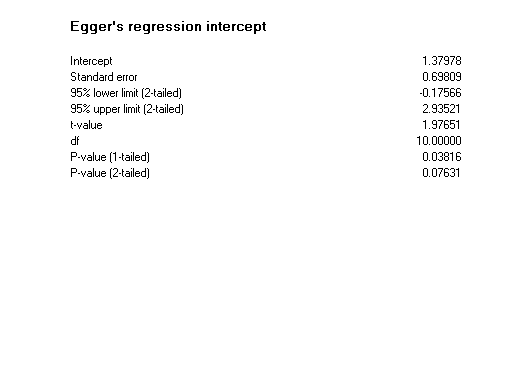


Funnel plot of trials reporting duration of PA at 24 months – continuous outcome measures

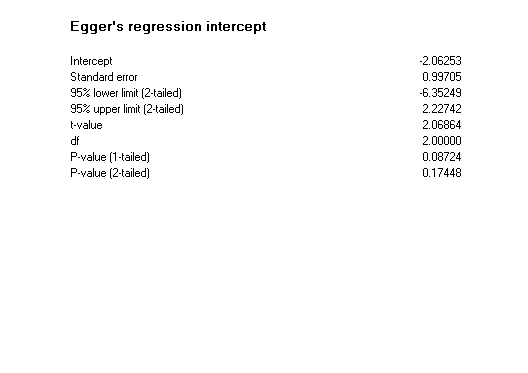

Supplement: Additional file 6 — Funnel plots and Egger's test results assessing publication bias in the meta-analyses. Funnel plots and Egger's tests were used to identify the presence of publication bias in the meta-analyses. Publication bias could be tested for in the meta-analysis of trials reporting pedometer step-counts (steps/day) at 12 months; duration of physical activity at 12 months - continuous outcome measures; and duration of physical activity at 24 months - continuous outcome measures. [file 1741-7015-11-75-S6.DOCX]
